# Allergy from infancy to adolescence. A population-based 18-year follow-up cohort

**DOI:** 10.1186/1471-2431-9-46

**Published:** 2009-07-25

**Authors:** Minna Kaila, Päivi Rautava, Doris Holmberg-Marttila, Tero Vahlberg, Minna Aromaa, Matti Sillanpää

**Affiliations:** 1 Paediatric Research Centre, University of Tampere and Tampere University Hospital, Tampere, Finland; 2 Clinical Research Centre, Turku University Hospital, Turku, Finland; 3 Centre of General Practice, Pirkanmaa Hospital District, Tampere, Finland; 4 Institute of Clinical Medicine, Biostatistics, University of Turku, Turku, Finland; 5 Turku City Hospital and Department of Public Health, University of Turku, Finland; 6 Departments of Public Health and Child Neurology, University of Turku, Turku, Finland

## Abstract

**Background:**

Anxious parents have many concerns about the future health of their atopic infants. Paediatricians and primary care practitioners need to seek knowledge on long-term outcomes in order to cope with the increasing caseload of suspected allergy and the concerns of parents. The aim of the study was to assess suspected and diagnosed allergy in infancy as predictors of allergy and asthma in adolescence.

**Methods:**

Families expecting their first baby and making their first visit to a maternity health care clinic in 1986 were selected as the study population in a random sample. There were 1278 eligible study families. The data were provided of the children at the ages of 9 and 18 months and 3, 5, 12, 15 and 18 years by health care professionals, parents, and adolescents (themselves).

**Results:**

At the age of 9 months, the prevalence of allergy suspicions was distinctly higher than that of allergy diagnoses. At the age of five years suspected allergy approaches were nil, and the prevalence of diagnosed allergy was about 9%. During the adolescence, the prevalence of self-reported allergy increases steadily up to the age of 18 years, and that of asthma remains at approximately 5%. Suspected allergy at the age of 9 or 18 months and at the 5 years of age does not predict allergy at adolescence. Compared with non-allergic children, children with definite allergy at the age of 5 were over 8 times more likely to have allergy and nearly 7 times more likely to have asthma in adolescence.

**Conclusion:**

An early ascertained diagnosis of allergy, but not suspicions of allergy, predicts prevailing allergy in adolescence. Efforts need to be focused on accurate diagnosis of early childhood allergies.

## Background

In recent years, the caseload related to suspected allergy and symptoms perceived as allergic in primary care have been increasing. Not all allergic symptoms persist, even though a longitudinal study indicated a direct relationship between childhood atopy and the development of asthma [1]. More severe atopy or atopy in early childhood increase the risk of respiratory symptoms compared to milder symptoms or later acquisition of atopy [2-4]. There is a multitude of known potential risk factors for the persistence or development of allergy and asthma [5]. Predicting occurrence of adult allergy and asthma in childhood is a challenge [1,6].

In Finland the prevalence of early childhood atopic eczemas (15–20%) is among the highest in the world [7]. Skin symptoms related to atopic eczema are even much more frequent (80%) [8]. Limited longitudinal follow-up data from childhood to adulthood are available about allergic diseases. Most prospective studies are short-term, with 2–3 years of follow-up. A third of children less than 2 years of age are suspected to have food allergy, but the majority of the suspicions dissolve over the following year [9]. In small children, the reported prevalence rate of atopic allergy is 13–17% [10], of food allergy about 8% [11] and of milk allergy about 2% [12,13]. One in three young adults is atopic [14]. In a Finnish 32-year follow-up study, the prevalence of adolescent asthma was 3–7%, and as many had asthmatic symptoms [15]. Recently, when the Finnish National Allergy Programme was launched [16], one of its goals was to increase tolerance of mild symptoms in children, and focus the efforts on improving the care of the more severe forms of the diseases.

The general aim of this study was to analyse the occurrence of suspected or diagnosed allergy and asthma from birth to early adulthood. The specific aim was to determine the risk of having allergy or asthma in adolescence after having diagnosed or suspected allergy at early childhood.

## Methods

The present study is part of the Finnish Family Competence (FFC) Study started in 1985 in the Province of Turku and Pori in South-Western Finland. Information was collected on the lifestyle and health behaviour of young Finnish families in order to further develop public health service.

The study cohort was randomly selected from two of the 21 central hospital regions of the country. Two central hospital regions were from South-Western Finland with a total population of 713,000 (15% of the population of Finland in 1985). Subject collection was based on stratified randomised cluster sampling. For stratification, the study area was divided into two parts, the southern area (Turku University Hospital Region) and the northern area (Satakunta Central Hospital Region). Each cluster consisted of municipalities in the sample health authority areas that did not differ significantly from all the other municipalities in the province [17]. Randomisation was carried out by selecting by lot 11 of the total of 35 health authority areas weighted for stratum. All 67 maternity health care clinics and 72 well-baby clinics of these 11 health authority areas participated in the study.

There is a public, countrywide network of well-baby clinics in Finland. Practically every child is followed up at regular intervals in these clinics. After the check-ups of midwife and neonatologist, the first visit to the well-baby clinic is scheduled a couple of weeks after birth with several regular follow-up visits during the first year of life. Thereafter the frequency of routine visits is reduced to once a year. The backbone of the well-baby clinics is the public health nurse together with the physician who both see the child and the family repeatedly.

Families expecting their first baby and having their first visit to a maternity health care clinic in 1986 were selected as the study population. About 99% of Finnish mothers use public maternity health care clinic services [18]. In the study area there were 1713 young families (i.e. nulliparous women and their husbands) expecting their first baby. Maternity health care nurses invited, according to the instructions, 1582 women to participate, and 1443 of them gave informed consent. The study subjects were married or cohabiting couples or single women (n = 36, 2.5%). Of the invited, 139 (8.8%) refused to participate. The occupational distribution of those who refused was similar to that of the participants [16]. There were 1294 deliveries, of which three were stillbirths, eight children died in infancy and five moved abroad. The number of the eligible families was thus 1278. The number of eligible study children was 1287, while seven women delivered twins and one triplet. To assess the representativeness of the results in the study population, several dropout analyses were conducted. Young mothers (age at the beginning of the study <20 years) (p < 0.02 in all study phases) and mothers with nine years basic education (p < 0.01 at 18 months and 12 and 15 years) dropped out significantly more often than others. No significant differences were found in allergic findings between study children who had complete data and those who dropped out. Hence, the study results can be generalized to cover well the whole study population.

**Figure 1:**
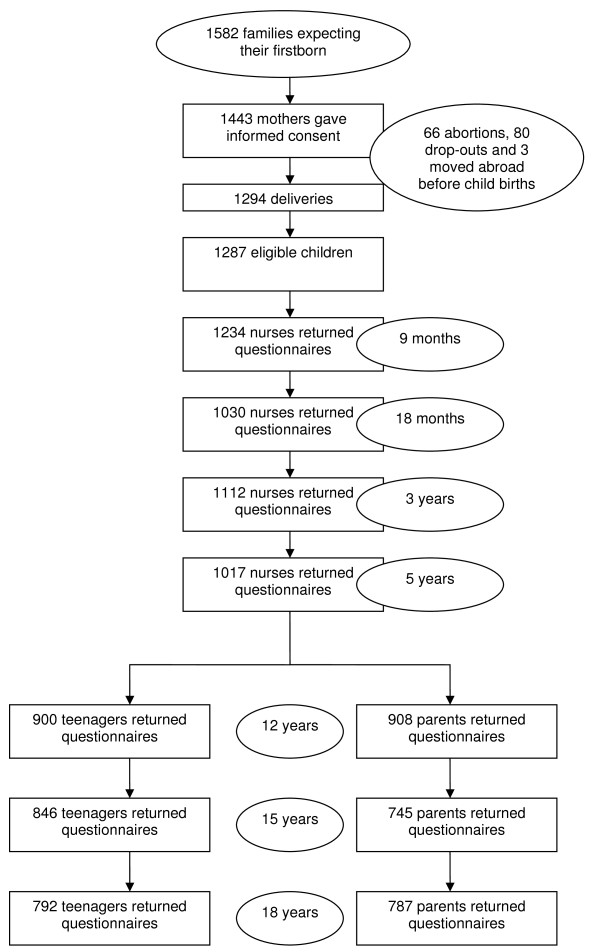
**Study participants**.

Data were collected from early pregnancy to the children's adolescence and adulthood, at first using questionnaires to the parents at routine visits to the maternity health care clinic during pregnancy, and thereafter at well-baby clinics after the child's birth, at the ages of 9 and 18 months, 3 and 5 years. At the age 12, 15 and 18 years, the questionnaires were completed at the study families' homes. At the ages of 9 and 18 months and 3 and 5 years, the data obtained from the files of the well-baby clinics were used. At the ages of 12, 15 and 18 years, the parents and children independently filled in and returned the completed questionnaires to the authors in sealed envelopes. Figure 1 shows the numbers of participants of the consecutive study phases. Furthermore, possible hospital care data were utilized for the study.

At the beginning of the follow-up, the mean age of the mothers was 25.4 years and that of the fathers 27.9 years. One percent of the mothers had completed less than 9 years of basic education, the others at least 9 years of basic education, and 42% of them had also completed 12 years of education (9 years of basic education and 3 years of upper secondary school).

### Allergy and asthma in early childhood

The individual health records maintained at the well-baby clinics were reviewed at the ages of 9 and 18 months, and 3 and 5 years, and categorized as follows: no allergy; allergy suspected by a physician and allergy diagnosed by a physician. The diagnosis was made by physicians (in Finland, only qualified physicians make diagnoses), recorded in the health record prospectively and the nurses collected this data from the health records carefully. Moreover, the nurses gathering the data from the health records were instructed to consider any such notes in the record when classifying as allergy suspicions (rashes, eczemas etc.).

### Allergy and asthma in adolescence

When the children were 12, 15 and 18 years of age, questions about allergy and asthma included in the Achenbach's Child Behaviour Check List (CBCL) [19] and the Youth Self Report (YSR) [20], were used. The parents and the adolescents were asked independently if 1) the child has allergy/I have allergy and 2) the child has asthma/I have asthma. The responses were categorized as follows: no fit (= unlikely allergy/asthma), some fit (possible allergy/asthma) and good fit (= definite allergy/asthma). The respondents were instructed to answer according to whether the claim (e.g. I have asthma) does or does not fit well to their particular situation. The agreement between the answers of the parents and the children was fairly good (the range of kappa coefficient values 0.5–0.8). The present paper is based on the YSR data received from the adolescents themselves.

### Statistical methods

The difference in prevalence between boys and girls was tested using the chi-squared test. The associations of diagnosed allergy (predictor variable) at the ages of 9 and 18 months, 3 and 5 years with definite self-reported allergy or asthma (dependent variable) at the ages of 12, 15 and 18 years were analysed using binary logistic regression with random intercept [21]. In the subject-specific model, which takes into account the correlation between repeated measurements at the ages of 12, 15 and 18 years, the repeated measurements of the dependent variable (definite allergy or asthma vs unlikely allergy or asthma) were analyzed using the same model. The predictive variable was divided into two categories, diagnosed allergy or no allergy. Separate models at different ages (9 and 18 months, 3 and 5 years) were used for the predictive variable. In addition, cumulative logistic regression was used to analyse the association of allergy or asthma (diagnosed, suspected or no allergy) at the ages of 9 and 18 months, 3 and 5 years with self-reported allergy or asthma (definite, possible or unlikely) at the ages of 12, 15 and 18 years. The results were quantified using odds ratios (ORs) or cumulative odds ratios (CORs) with 95% confidence intervals (95% CI). P-values lower than 0.05 were considered statistically significant. The analyses were done with PROC GLIMMIX and PROC LOGISTIC of the SAS System for Windows, release 9.1.3 (SAS Institute, Cary, NC, USA).

The study design was approved by the Ethical Committee of the Turku University's Faculty of Medicine.

## Results

The prevalence of suspected and diagnosed allergy in children under 5 years of age according to the well-baby clinic health records, and the prevalence of physician diagnosed allergy are presented in Table 1. The prevalence of self-reported allergy and asthma during adolescence are given in Table 2. At the age of 9 months, the prevalence of allergy suspicions was distinctly higher than that of allergy diagnoses. There is a peak in the prevalence of suspected allergy at 18 months and of diagnosed allergy in children less than 3 years of age. At the age of five, the number of suspected allergy approaches nil, and the prevalence of diagnosed allergy is about 9%. There is a steadily growing trend up to age 18 years in the self-reported prevalence of allergy in adolescence. The prevalence of asthma is more stable and stays approximately at the level of 5%.

**Table 1:** The prevalence, n (%), of suspected allergy cases and diagnoses in children under 5 years of age reported by well-baby clinic nurses

Nurse-reported allergy	9 months (n = 1234)		18 months (n = 1030)		3 years (n = 1112)		5 years (n = 1017)	
Suspected	151	(12.2)	142	(13.8)	104	(9.3)	8	(0.8)
Diagnosed	78	(6.3)	115	(11.2)	132	(11.9)	91	(8.9)
No allergy	1005	(81.5)	773	(75.0)	876	(78.8)	918	(90.3)

**Table 2:** The prevalence, n (%), of self-reported allergy and asthma during adolescence years

Self-reported allergy	12 years n = 880		15 years (n = 832)		18 years (n = 785)	
Possible	171	(19.4)	161	(19.3)	184	(23.4)
Definite	117	(13.3)	136	(16.4)	148	(18.9)
Unlikely	592	(67.3)	535	(64.3)	453	(57.7)

Self-reported astma	12 years n = 891		15 years n = 840		18 years n = 789	

Possible	24	(2.7)	45	(5.4)	47	(6.0)

Definite	51	(5.7)	44	(5.2)	42	(5.3)

Unlikely	816	(91.6)	751	(89.4)	700	(88.7)

The older the child the more accurately the diagnosed and even suspected allergy predicts self-reported allergy in adolescence (Table 3). Suspected allergy at 9 or 18 months and at 5 years of age does not predict allergy in adolescence. By contrast, the risk of allergy in adolescence is 2.9 – 4.1-fold if the child has been diagnosed as having allergy at the age of 3 years and 3.4 – 6.3-fold with allergy diagnosed at the age of 5 years. Allergy diagnosis in childhood tends to increase the risk of self-reported adolescent asthma. Allergy suspicions in early childhood play no predictive role for adolescent asthma.

**Table 3:** Medically suspected and/or diagnosed allergy at the ages of 9 and 18 months, 3 and 5 years predictive of self-reported allergy and asthma at the ages of 12, 15 and 18 years.

		Diagnosed allergy		
Age	Allergy	12 y COR (95% CI)	15 y COR (95% CI)	18 y COR (95% CI)
9 months	Suspected	1.4 (0.95 – 2.1)	1.4 (0.9 – 2.1)	1.5 (0.96 – 2.2)
	Diagnosed	2.8 (1.7 – 4.7)	2.3 (1.4 – 3.9)	1.8 (1.1 – 3.2)
				
18 months	Suspected	1.9 (1.3 – 2.8)	1.5 (0.96 – 2.2)	1.4 (0.96 – 2.2)
	Diagnosed	1.9 (1.2 – 3.0)	1.7 (1.1 – 2.6)	1.3 (0.8 – 2.1)
				
3 years	Suspected	1.9 (1.2 – 3.0)	2.0 (1.3 – 3.3)	2.0 (1.3 – 3.2)
	Diagnosed	4.1 (2.3 – 6.1)	3.0 (2.0 – 4.6)	2.9 (1.9 – 4.4)
				
5 years	Suspected	2.1 (0.6 – 8.1)	0.3 (0.0 – 2.6)	1.0 (0.2 – 4.5)
	Diagnosed	6.3 (3.9 – 10.2)	4.1 (2.5 – 6.8)	3.4 (2.0 – 5.6)

		Asthma		

Age	Allergy	12 y COR (95% CI)	15 y COR (95% CI)	18 y COR (95% CI)

9 months	Suspected	1.3 (0.7 – 2.6)	1.4 (0.7 – 2.7)	1.5 (0.8 – 2.7)

	Diagnosed	2.5 (1.2 – 5.3)	2.1 (1.04 – 4.4)	2.0 (0.9 – 4.3)

				

18 months	Suspected	1.9 (0.99 – 3.6)	1.7 (0.9 – 3.1)	1.6 (0.8 – 3.0)

	Diagnosed	2.1 (1.02 – 4.2)	1.4 (0.7 – 2.9)	1.4 (0.7 – 3.0)

				

3 years	Suspected	1.5 (0.7 – 3.5)	1.9 (0.9 – 3.8)	2.6 (1.2 – 4.4)

	Diagnosed	4.7 (2.7 – 8.3)	3.3 (1.9 – 5.7)	2.8 (1.5 – 5.0)

				

5 years	Suspected	1.9 (0.2 – 17.2)	3.5 (0.6 – 19.3)	3.2 (0.6 – 17.7)

	Diagnosed	6.7 (3.7 – 12.2)	5.3 (3.0 – 9.5)	4.9 (2.7 – 8.9)

The analysis is based on logistic regression with random intercept (COR95%CI = cumulative odds ratio with 95% confidence intervals)

**Figure 2:**
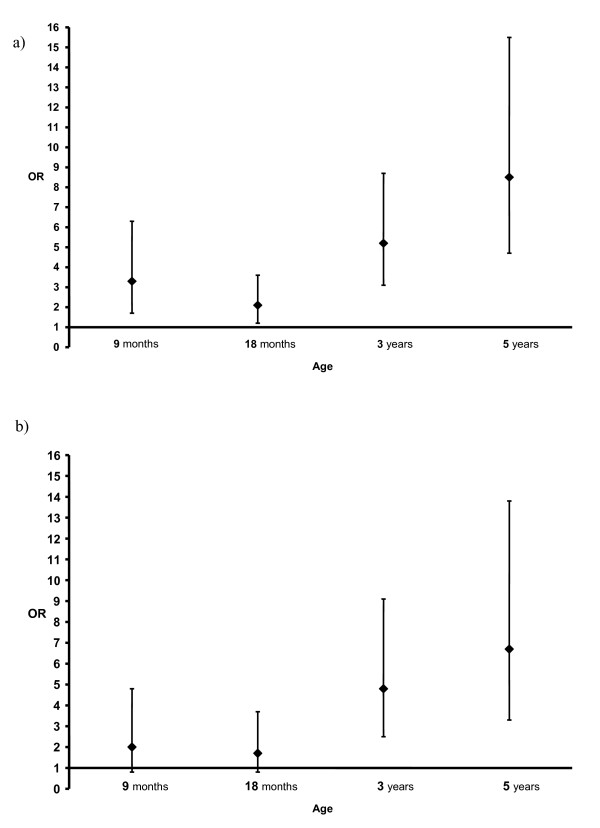
**The subject-specific association of allergy at the ages of 9 and 18 months, 3 and 5 years with self-reported allergy, and allergy (a) and asthma (b) at the ages of 12, 15 and 18 years**. In the subject-specific model, analysis takes into account the correlation structure of the repeated measurements, that is, whether a same child who has allergy at 9 months, 18 months, 3 and 5 years, has allergy (a) or asthma (b) at the ages 12,15, and 18 years. The predictive variable was divided into two categories, diagnosed allergy or no allergy at ages of 9 and 18 months, 3 and 5 years. The analysis is based on logistic regression with random intercept, and the results are presented as Odds Ratios (OR) with 95% Confidence Intervals.

Figure 2 shows the individual risk of a definitely allergic child to become an allergic or asthmatic adolescent. Analysis takes into account the correlation structure of the repeated measurements, that is, whether the same child who has allergy at 9 months, 18 months, 3 and 5 years has allergy or asthma at the ages 12,15, and 18 years. It shows that if a child has a diagnosis of allergy at the age of 5 years, there is a high risk of allergy (OR = 8.5, 95% CI 4.7 – 15.5) and asthma (OR = 6.7, 95% CI 3.3 – 13.8) at the age of 18 years. This model also shows that the older the child when allergy was diagnosed, the more accurately this predicts allergy or asthma in adolescence.

## Discussion

The main finding of this study was that suspected allergy in early childhood does not predict later allergy diagnoses. This stresses the importance of a careful diagnosis, since definitely diagnosed, but not suspected allergy at the ages 9 and 18 months and 3 and 5 years does predict allergy at adolescence. This association becomes more apparent the later the allergy diagnosis is confirmed. Diagnosed allergy at the age of 3 and 5 years even predicted asthma in adolescence.

The original study population was highly representative of the target population. Follow-up of the study sample was, thanks to the good coverage of the Finnish maternity and well-baby clinic network, very successful with good participation rates throughout the study period [17]. Thus, the validity of the study design can be considered very satisfactory. The main limitation of this study is that the prevalence of adolescent allergy-like indicators and asthma were based on adolescents' self-reports; however, with repeated use of the same questionnaires. Since parents' reports did not significantly differ from adolescents' self-reports, self-reports were used. The presence or absence of early childhood diagnoses of allergies and asthma were based on the notes in the individual health records, from where the data were collected by health care professionals. The diagnoses were made by physicians. Testing the intra-individual variability of different physicians in 72 well baby clinics was not possible but they worked according to the general diagnostic criteria of the time. Data collection was structured, systematic and the same methods were used throughout as appropriate.

Consistent with the findings of other studies [1,22-26], we found that an allergy diagnosis in childhood is a significant risk factor for adolescent allergy and asthma. In the present study, subjects with diagnosed allergy at the age of 5 years were over 8 times more likely to have allergy and nearly 7 times more likely to have asthma than healthy subjects. Of note is that suspected allergy before the age of 2 years had no significant effect on the prognosis of allergy or asthma. However, a definite allergy diagnosed at the age of 9 and 18 months significantly predicted the occurrence of allergy at the age of 12 and 15 years but did not significantly predict asthma at the same ages. Thus, the risk of developing allergy or asthma in adolescence was related to the age when a definite allergy was diagnosed. This could be connected with the previous finding that the older the child the more reliable the food allergy diagnosis [27]. Severe atopy in childhood has shown to be a major determinant of the prognosis of allergic asthma in adulthood [26]. The peak prevalence of suspected allergies, about 14%, was seen at age less than 2 years, whereas at age 5 years there were less than 1% suspected and 9% diagnosed allergies.

Anxious parents have many concerns about the future health of their atopic infants [28]. The well-baby clinic professionals are at present dealing with increased public awareness of allergic diseases and with recurrent queries about very mild and passing symptoms [29]. The definition and diagnosis of allergies in early childhood remain challenging because of lack of uniform criteria and availability of objective tests to support diagnosis. Mild allergies, especially, constitute a diagnostic problem [29]. These clinical challenges will be tackled during the years of the National Allergy Programme and much emphasis will be put on accurate diagnosis [16]. Specifically, one main goal is to halve the prevalence of elimination diets by 2018. The results of the present study support the notion of putting emphasis on accurately diagnosing early childhood allergies, since suspicions without diagnosis do not seem to indicate later allergologic problems. Most children developing early atopic symptoms would not have been considered having high atopy risk at birth [30]. The prevalence rates of suspected and diagnosed allergy in early childhood years were in line with the results of previous studies [10]. During adolescent years, the prevalence of allergy increased from 13% at the age of 12 years to 19% at the age of 18 years. These prevalence of allergy confirm those from previous studies. The prevalence of adolescent asthma, about 5%, is also concordant with previous results from Finland [7].

## Conclusion

The clinical implications of the present results seem clear and support the conclusions and lines of action of the Finnish Allergy Programme. Suspected allergies in the early years of life do not pose a threat to the infants' future well-being – and the need for accurate diagnostic procedures is clear. Paediatricians and primary care professionals can reassure young families that allergy-type symptoms most likely will pass, especially if they are mild. Vice versa, with a definite diagnosis of allergy at the age of 5 the child will probably remain allergic or asthmatic in his/her teens.

## Abbreviations

CBCL: the Achenbach's Child Behaviour Check List; FFC: the Finnish Family Competence Study; YSR: the Youth Self Report.

## Competing interests

The authors declare that we have no competing interests. The authors have no institutional or corporate affiliations.

## Authors' contributions

Pediatrician MK, a child allergologist, contributed to the study design, the analysis and interpretation of data in the prevention and progress of the allergies. Pediatrician PR, as a specialist of epidemiological studies and preventive medicine in well-baby clinic setting, made contributions to the design, the acquisition of data, analysis, and interpretation of data. Dr DHM, a specialist of primary health care, contributed to the design, analysis, and interpretation of data. Biostatistician TV made contributions to the design of the study and its' statistical analyses, and interpretation of the statistical text. Pediatrician MA, a specialist of out-patient paediatrician, made contributions to the epidemiological studies in the design, the acquisition of data, analysis, and in the interpretation of data. A specialist of long term follow-up studies and epidemiological studies, professor of child neurology MS made contributions to the design, the acquisition of data, analysis, and interpretation of data. All authors have read and approved the final manuscript.

## Pre-publication history

The pre-publication history for this paper can be accessed here:


